# Advances in Circular RNA and Its Applications

**DOI:** 10.7150/ijms.71840

**Published:** 2022-05-27

**Authors:** Xian Zhao, Youxiu Zhong, Xudong Wang, Jiuheng Shen, Wenlin An

**Affiliations:** National Vaccine & Serum Institute (NVSI), China National Biotech Group (CNBG), No. 38 Jing Hai Second Road, Beijing, 101111, China

**Keywords:** circRNA, miRNA, mRNA, back-splicing, circRNA-based therapeutics

## Abstract

Circular RNA (circRNA) is a novel endogenous non-coding RNA (ncRNA) that, like microRNA (miRNA), is a rapidly emerging RNA research topic. CircRNA, unlike traditional linear RNAs (which have 5' and 3' ends), has a closed-loop structure that is unaffected by RNA exonucleases. Thus, circRNA has sustained expression and is less sensitive to degradation. Since circRNAs have many miRNAs binding sites, eliminating their repressive effects on their target genes can strongly enhance their expression. CircRNAs serve an important regulatory role in disease onset and progression via specific circRNA-miRNA interactions. We summarized the current progress in elucidating mechanisms and biogenesis of circRNAs in this review. In particular, circRNAs can function mainly as miRNA sponges, regulating host gene expression and protein transportation. Finally, we discussed the application prospects and significant challenges for the development of circRNA-based therapeutics.

## Introduction

CircRNA, a novel non-coding RNA, is produced by back-splicing of mRNA precursors (pre-mRNAs) 1-5. Sanger et al.^6^ have discovered pathogenic single-stranded cyclic-like viruses in some higher plants in 1976, which was the first discovery of covalently closed circRNA in humans. Since then, circRNAs have been discovered successively in yeast mitochondria 7, hepatitis delta virus (HDV) 8, human E-twenty-six-1 (ETS-1) gene 9, 10, mouse sex-determining region Y (Sry) 11, rat cytochrome P450 2C24 gene 12 and human P450 2C18 gene 13. Although circRNAs have been discovered for several decades, they were considered to be a by-product of mis-splicing for a long time. CircRNA harbors a covalently closed loop structure without 5' caps and 3' tails, and it is formed by reverse splicing. This is an extremely rare phenomenon in nature and even treated as a genetic accident or an operational error. Therefore, circRNAs have not received enough attention.

Several circRNAs have been identified because of rapid advances in high-throughput RNA sequencing and bioinformatic analysis. In 2012, Salzman and co-workers 14 first suggested that pre-mRNAs can form circRNAs through reverse splicing. These circRNAs are present in a variety of cell types in humans. Since then, researchers have focused on the function of circRNAs, and Nature reported two articles on circRNAs in 2013. Hansen and co-workers 15 found that circRNA can block miR-7, indicating that circRNAs can act as “miRNA sponges”. Memczak et al. 16 found abundant stable circRNAs in human, mouse and nematode. Also, researchers have discovered the mechanism of circRNAs formation. More than 25,000 specific RNA types were identified in human fibroblasts by Jeck and co-workers 1, revealing that circRNAs are produced by exon skipping. They confirmed the role of ALU repeats in circRNAs formation. Zhang et al. 17 also found that intron circularization can also produce circRNAs. Since then, the study of circRNAs has rapidly become a research focus.

CircRNAs, unlike traditional linear mRNAs, contain a closed-loop structure and lack free ends. This structure is more stable and conserved than linear RNA. This review has highlighted the latest progress on the formation mechanisms and main functions of circRNAs. Also, possible research trends of circRNAs in the future are forecasted.

## 1. Biogenesis and functions of circRNAs

CircRNAs are covalently closed RNA molecules formed by reverse splicing of pre-mRNAs through exons or introns, and only exon-derived circular RNAs (EcircRNAs) are mostly found in the cytoplasm 1, while intron-derived circular RNAs (ciRNAs) are mainly located in the nucleus 17. It was also found that certain circularized exons retain introns, and such circRNAs are called exon-intron circRNA (EIciRNA) and are mainly detected in the nucleus 18. The formation mechanism of circRNA is not completely clear, only three models have been reported: intron-pairing-driven circularization, RNA-binding protein (RBP) -mediated circularization, and lariat-driven circularization.

### 1.1 Biogenesis of circRNAs

#### 1.1.1 Intron-pairing-driven circularization

**Fig 1:**
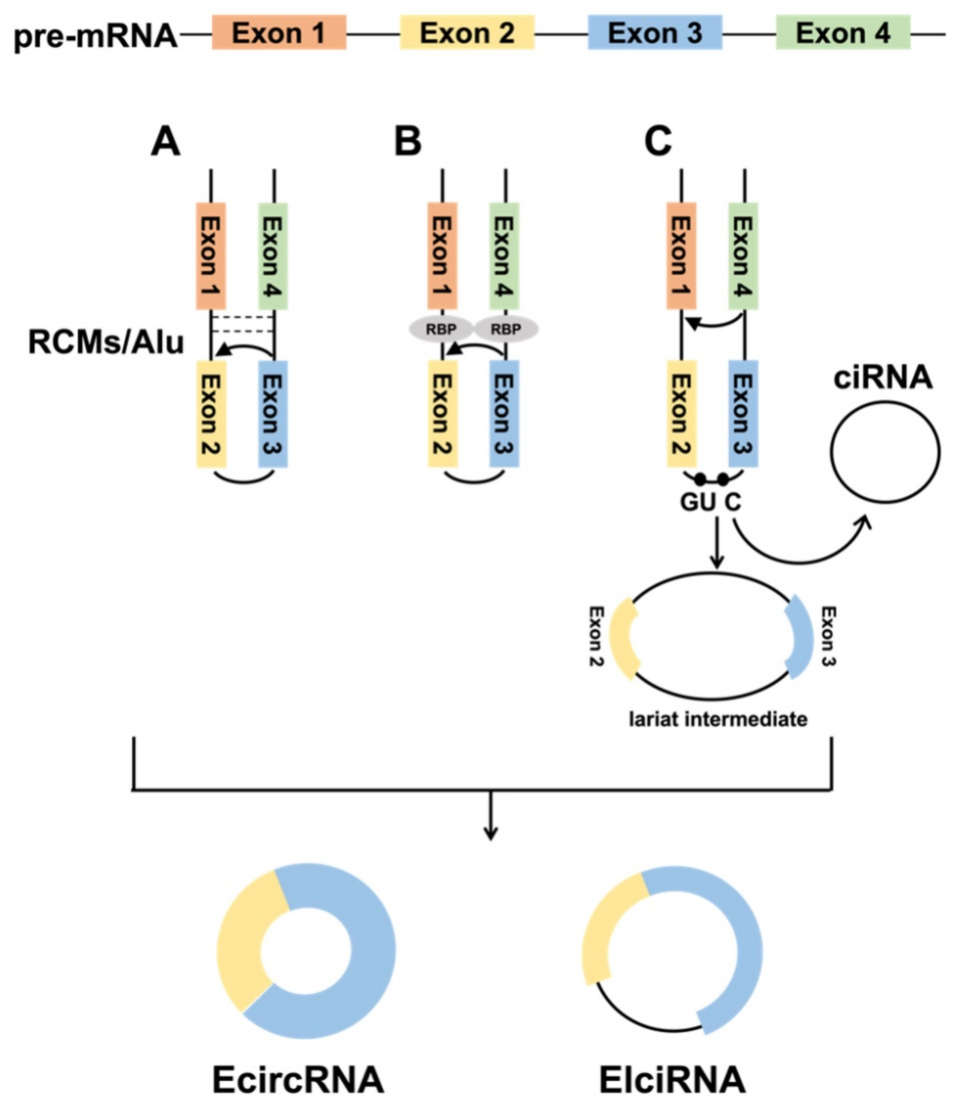
Biogenesis of circRNAs. **A.** The back-splicing circularization requires the help of the complementary sequences (ALU repeats and RCMs). **B.** RBP-mediated circularization. **C.** Lariat-driven circularization.

Due to complementary base pairing of intron sequences on both sides of the circularized exon, the 5' splice site can directly join with the 3' splice site to form a circRNA. The primate-specific ALU repeats have more than 1 million copies in the genome, accounting for over 10% of the human genome. Jeck et al. 1 found that ALU repeats are the main way of circularization in human genes. However, ALU repeats are only present in a small fraction of vertebrates, and Capel et al. 11 identified up to 15,000 nt of nearly complete complementary pairs of intron sequences in circSRY, and these reverse complementary matches (RCMs) promote the formation of hairpin structures in transcripts, thus facilitating the SRY circularization. Ivanov et al.^19^ analyzed 300 to 1,100 ecircRNAs of *Caenorhabditis elegans* (*C. elegans*) and found that introns were rich in RCMs and significantly higher than mRNAs, strongly demonstrating that RCM provides essential functions in circRNA formation (Fig. 1A). Meanwhile, computer modeling successfully verified that the analysis of RCMs in introns could successfully predict the circRNA.

#### 1.1.2 RBP-mediated circularization

RBPs are key to promoting tissue-specific circRNA formation and can be involved in circRNA formation by binding specific motifs in flanking intron sequences (Fig. 1B). For example, the ALU repeats is the major binding target site of adenosine deaminase 1 acting on RNA (ADAR1). ADAR1 inhibits circRNA formation by destabilizing paired elements such as ALU repeats via A→I RNA editing in double-stranded RNA (dsRNA) substrates, which converts adenosines to inosines 20.Conn et al. screened quaking (QKI) proteins from a great number of binding proteins that promote circRNA formation, and QKI promotes exon circularization by binding to the ACUAACN1-20UAAC sequence 21. In humans and *Drosophila*, the second exon of the splicing factor *muscleblind* (MBL) promotes its circularization to form circMbl, which can join with MBL to reduce the amount of MBL, eventually reducing circMbl synthesis 22. Although RBPs promote the biogenesis of circRNAs, the inhibitory effect of RBPs on circularization has also been reported. As an example, DEAH-box helicase 9 (DHX9) controls the circRNA formation by downregulating ALU repeats-induced intron pairing. In contrast, removal of DHX9 increases circRNA content 23.

#### 1.1.3 Lariat-driven circularization

When pre-mRNAs undergo classical GU/AG splicing 24, exon skipping 25 can occur, producing a lariat intermediate containing intron-exon, which subsequently undergoes reverse splicing to form a circRNA (Fig. 1C). Introns are usually debranched and degraded during pre-mRNA splicing, but those with a 7-nt GU-rich element and an 11-nt C-rich element can form ciRNAs 26. Various studies have indicated that this lariat structure is also widespread in plants 27.

### 1.2 Biological functions of circRNAs

CircRNA was previously regarded as a splicing by-product, thus it was considered incidental and of no biological significance. Further in-depth studies have demonstrated that circRNA is not a splicing by-product, but is widely sourced, stable, conserved, tissue-specific, and plays many potential functions in living organisms 28, 29. Here, we summarized the known functions of circRNAs, such as acting as miRNA sponges, regulating transcription, transporting proteins, and facilitating protein-protein interactions.

#### 1.2.1 Acting as miRNA sponges

**Fig 2:**
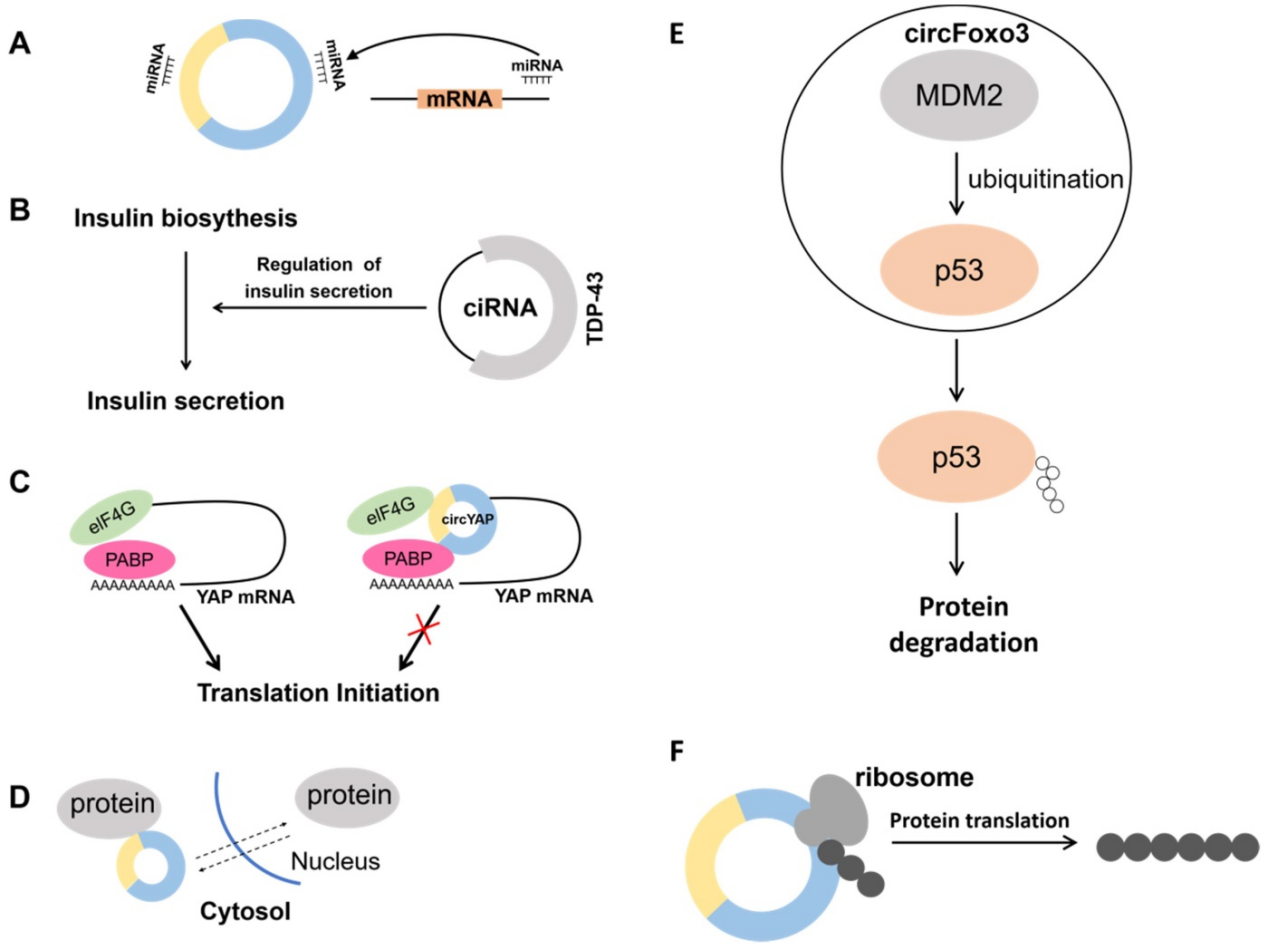
Biological functions of circRNAs. **A.** Acting as a miRNA sponge.** B.** In β-cells, ciRNA interacts with the RBP TDP-43 and controls the expression of genes necessary for insulin release. **C.** The circYap could bind with Yap mRNA, eIF4G and PABP simultaneously. Inhibiting this interaction represses the translation initiation of Yap. **D.** Regulation of nucleocytoplasmic transport. **E.** MDM2, an E3 ubiquitin-ligase, targets p53 for proteasome-dependent degradation. CircFoxo3 enhances the interaction between MDM2 and p53, and further promotes the poly-ubiquitination and degradation of p53. **F.** Translating to protein in a cap-independent manner.

Since circRNAs contain miRNA binding sites, they can function as miRNA sponges to regulate miRNA expression (Fig. 2A) 30. MBL can promote the synthesis of circMbl and there are specific MBL binding sites on the synthesized circMbl. When MBL is highly expressed, it promotes circMbl formation and suppresses the expression of linear transcripts. The research revealed that circRNAs act as miRNA sponges in bladder cancer, where the expression of circ-ITCH is lower in bladder cancer tissues than that of in normal tissues. Moreover, studies have indicated that circ-ITCH binds to miR-17 and miR-224 to act as a miRNA sponge, inhibiting the development of bladder cancer through direct regulation of p21 and PTEN 31. High expression of circ-TFRC was also detected in bladder cancer patients, and the study showed that circ-TFRC combined with miR-107 exerted a spongy effect and promoted the development of bladder cancer 32. Analysis of tissue samples from gastric cancer patients revealed a significant decrease in the expression of circCCDC9, and it was shown that circCCDC9 combined with miR-6792-3p acted as a sponge thereby inhibiting the development of gastric cancer through regulation of CAV1 33. CircRNA also plays a key role in kidney cancer. Bioinformatics data revealed that circSDHC was highly expressed in kidney cancer patients. Meanwhile, it was shown that circSDHC binds to miR-127-3p to play a spongy role thereby regulating CDKN3/E2F1 to promote the development of kidney cancer 34. That is, all these studies provide potential targets for cancer therapy.

#### 1.2.2 Regulating transcription

In addition, despite being involved in regulating host genes expression as miRNA or protein sponges, circRNA can also be involved in transcriptional regulation. A circRNA generated from the insulin gene was recently discovered to interact with RBP TDP-43 and play a critical role in controlling the transcription of insulin secretion-related genes. It is well-known that pancreatic β-cells secrete insulin and insufficient insulin secretion triggers diabetes. It was shown that the second intron of the insulin gene forms ciRNA, which affects insulin secretion by interacting with the RBP TDP-43 (Fig. 2B) 35. It was demonstrated that the RBP HuR can bind not only to hundreds of coding and non-coding mRNAs 36, 37, but also to circRNAs to achieve the effect of regulating transcription. CircPABPN1 can bind to HuR thereby reducing the binding of HuR to PABPN1 mRNA and ultimately inhibiting its translation 38. In addition, circRNA can also bind to its corresponding mRNA to affect its translation process. Because Yap is a critical component of the Hippo pathway, it plays an important role in tumorigenesis. Overexpression of Yap corresponding circRNA in cancer cells significantly reduced the amount of Yap protein, but did not alter its mRNA level. It was found that circYap can bind both its corresponding mRNA, translation initiation protein eIF4G and PABP, blocking the interaction of PABP with the mRNA 3' tail and eIF4G with the 5' cap, thus blocking the translation process (Fig. 2C) 39. Therefore, it is essential to analyze the action site of the circRNA with mRNA. Blocking the translation process may reduce the expression of proteins and thus inhibit cancer initiation and progression.

#### 1.2.3 Protein Translocation

CircRNA can affect protein function by translocating proteins between the nucleus and cytoplasm (Fig. 2D). For example, CircFoxo3 is highly expressed in the hearts of aged mice and patients. It can bind multiple proteins such as ID-1, E2F1, HIF1α and FAK and no longer exert its anti-aging and anti-stress effects by promoting the retention of these proteins in the cytoplasm, leading to increased cellular senescence 40. In contrast, studies have indicated that highly expressed CircAmotl1 in samples from tumor patients assists the oncogenic c-MYC (MYC) transcription factor to translocate to the nucleus, and the elevated amount of *c-myc* in the nucleus also improves its stability and target binding capacity 41. Meanwhile, a large amount of CircAmotl1 was found in neonatal heart tissues, and it was demonstrated that CircAmotl1 can bind and promote AKT1 phosphorylation, transport PDK1 and AKT1 into cells, and play a role in inhibiting apoptosis and promoting cardiac repair 42. Circ-Amotl1 also has the efficacy of promoting wound repair. Circ-Amotl1 binds to STAT3 and transports it into the nucleus to enhance the expression of fibronectin through the regulation of Dnmt3a and miR-17 43.

#### 1.2.4 Facilitating protein-protein interactions

Previous studies have found that circRNAs can function as protein scaffolds to promote protein-protein interactions. Proteins do not exist in isolation but have specific three-dimensional spatial structures. Protein-protein interactions perform functions that are important for regulating cells and their signaling pathway. For example, the E3 ubiquitin ligase MDM2 can induce both p53 and Foxo3 ubiquitination, and the ubiquitinated p53 and Foxo3 will be degraded by the proteasome. CircFoxo3, p53 and MDM2 forms a complex that promotes MDM2-induced p53 ubiquitination, which in turn promotes p53 degradation. Conversely, Foxo3 cannot be ubiquitinated because the formation of a complex composed of Foxo3, MDM2 and circFoxo3. This also results in elevated PUMA expression, leading to apoptosis (Fig. 2E) 44. This study demonstrates that circRNAs can promote protein-protein interactions. However, the analysis of circRNAs nucleotide sequences is not enough to predict circRNAs-protein interactions. Because circRNA three-dimensional structure may severely affect their protein binding capacity.

#### 1.2.5 Protein translation

CircRNAs can not only control transcription, but also can be translated to generate proteins (Fig. 2F). Yang and co-workers 45 demonstrated that a great number of m6A motifs exist on circRNA in human cells, and a single m6A site is sufficient to initiate translation of circRNA with the participation of multiple proteins. Not only CircRNA can be translated by m6A modification, but circRNA containing internal ribosomal entry site (IRES) can also be translated in vivo or in vitro 46, for example, circMbl3 47 and circ-ZNF609 48 can be translated into proteins by IRES. Wu et al. 34 found significantly elevated expression levels of circ-SMO in gliomas. Circ-SMO encoded a novel protein named SMO-193aa, and overexpression of SMO-193aa promoted the activation of the Hedgehog pathway, thereby promoting tumor progression in gliomas. Legnini et al. 48 used a genetic screen to investigate muscle differentiation. The function of circRNA was found to specifically regulate myogenic cell proliferation. Circ-ZNF609 is formed by circularization starting from the second exon of the ZNF609 gene and contains a 753-nt open reading frame (ORF) from the start site to the termination site. This study also verified that Circ-ZNF609 can be translated Peng et al. 49 , on the other hand, found that CircAXIN1 could encode the novel protein AXIN1-295aa and promote the development of gastric cancer through the Wnt pathway. In addition, the special structure of circRNA allows it to be translated by rolling circle translation to produce proteins of different sizes. Abe et al. 50, 51 found that circRNA containing multiple ORFs without stop codons could be translated in E. coli and human cells. This process is most similar to rolling circle amplification (RCA). The above studies demonstrate that under certain conditions circRNAs can be translated into proteins with divergent biological functions.

## 2. Approaches to sequence analysis of circRNAs

### 2.1 Two strategies for circRNAs library construction

Early conventional RNA-Seq library construction methods can only capture a small amount of circRNA. Currently, there are two library construction methods for circRNA. The one is to construct the circRNA library without polyA screening and transcriptome sequencing after removing rRNA. The other is to generate the library with rRNA removal in combination with RNase R treatment. However, some linear transcripts that do not contain polyA tails and are not completely degraded after RNase R treatment usually interfere with the downstream analysis of circRNA. To address this issue, Pandey et al. 52 identified highly enriched circRNA using a novel method “RNase R treatment followed Poly(A) tailing and poly(A) Depletion” (RPAD).

### 2.2 CircRNAs sequencing and data analysis

As significant amounts of circRNA have been identified, there is an urgent need to establish tremendous number of databases for storing these circRNAs data. Table 1 lists the updated databases for circRNA, which include both commonly used circRNAs databases and recently established databases. Among those, the riboCIRC database is a database resource on circRNAs translation after TransCirc. RiboCIRC is a translation group data-oriented database that explores 3,168 existing ribo-seq sets and 1,970 paired RNA-seq sets, encompassing 314 studies and 21 species. Approximately more than 2,000 circRNAs with translational potential were captured by paired Ribo-seq/RNA-seq. Like TransCirc, riboCirc integrates many translation-related information, and circRNAs captured by ribo-seq are not necessarily in translation, so all results of riboCIRC are subject to further experimental validation.

The cancer-specific circRNA database CSCD2 is a completely new version of CSCD 53. This database collected a large amount of human cancer-related transcriptome sequencing data, which was further analyzed and integrated to be developed into a cancer-specific circRNA integrative interaction database, providing a new resource platform for exploring the potential functions of circRNAs in cancer research. MiOncoCirc is a clinical tumor sample-based circRNA database that can be used for cancer biomarker development.

Furthermore, VirusCircBase is the first database resource for viral circRNA, which contains 11,924 circRNA of viral origin, covering 23 viruses in four major classes.

## 3. Challenges and copings of circRNAs technology

The development of circRNAs therapeutics is still in its nascent stage, and there are still multiple challenges to overcome, among which, the three main challenges are (1) design and optimization of circRNAs, (2) circularization efficiency of circRNAs, and (3) chemical manufacturing and control (CMC) process development of circRNAs.

### 3.1 CircRNA design and optimization

Biological function and mechanism of circRNA indicate that targeting disease-related genes could be a therapeutic approach. Therefore, circRNA manipulation is highly desired 5. A lot of efforts have been paid into the design and optimization of circRNA. Meganck et al. 69 developed a circRNAs overexpression vector based on adeno-associated viral vector (rAAV), which uses CMV promoter with polyA signal sequence of SV40. However, the disadvantage is that it can cause immune rejection. In addition, the intron sequence of *ZKSCAN1* and *HIPK3* genes forming the circRNAs was used to construct the circRNAs expression system 70. In this system, the ORF of the split GFP was used to verify that the expressed circRNAs could translate the protein after circularization. Only the correct circRNAs could form the ORF of the complete GFP and express the GFP protein, and this expression system was used in a variety of in vivo tissues. Litke et al. 71 used a self-shearing nuclease for upstream and downstream cleavage of the sequence to be circularized. This circularization process was achieved with the endogenous RNA ligase RtcB. The problem, however, is that the final product contained the sequence to be circularized as well as the ligated stem sequence carried for circularization.

Recently, circRNAs overexpression vectors such as pLCDH-ciR or pCD-ciR carry optimized flanking loop-forming frameworks containing modified binding sites for RBPs such as ALU repeats and QKI, and use newly designed circularization-mediating sequences to ensure accurate and efficient circularization of inserted circRNA. Although there have been many attempts and successes in the design and optimization of circRNAs, there are still a lot of problems need to be addressed.

### 3.2 Circularization efficiency and expression efficiency of circRNAs

Due to the special structure of the circRNAs, it has the problem of not being able to form a loop or not having high circularization efficiency. Special sequences and complex secondary structures may affect circularization efficiency. Although many institutions have studied the translation mechanism of circRNA for years, many of these mechanisms remain unsolved. Further analysis of these mechanisms will help to better design circRNA and improve their circularization efficiency.

As mentioned above, pre- and mature linear RNAs are commonly used in overexpression approaches. Furthermore, substituting a weak promoter with a strong one can promote circRNA products. However, this alteration can increase the formation of both linear and circular RNAs. RBPs including MBL 22 and Quaking (QKI) 21 may also help to stimulate circRNA synthesis. The issue is that the binding sites are required.

Artificial modulatory techniques for circRNA biogenesis have recently been created with great success. The sequence-specific RNA binding domains of human Pumilio1 (PUF domain) are combined with functional domains that can create engineering circRNA regulators (ECRRs). ECRRs not only can stimulate the biogenesis of exogenous circRNA reporters, but also enhance the creation of endogenous circRNA 72.

### 3.3 CMC development

The CMC process for circRNA is divided into four steps: (1) plasmid construction and proof of concept (2) in vitro synthesis and purification (3) circularization (spliceosomes and ligases) and purification (4) encapsulation and partitioning. Although there are several different ways to prepare circRNAs, there are no mature processes and facilities for large-scale production of circRNAs at this stage, which limits circRNAs-oriented drug development. The mainstream technology for the preparation of circRNAs is mainly *in vitro* transcription followed by self-shearing to form circRNAs.

circRNAs overexpression vectors carry optimized flanking loop-forming frameworks containing modified binding sites for RBPs such as ALU repeats and QKI, and use newly designed circularization-mediating sequences to ensure accurate and efficient circularization of inserted circRNA. However, these sequences contain irrelevant sequences and affect the natural structure and unique function of circRNAs. It is required to dedicatedly design and optimize the sequences of the circularization junctions.

In addition, the use of ligase for the ligation of RNA will generate a considerable amount of polymerization by-products, which makes the actual synthesis and subsequent product purification and recovery process more complicated, leading to low preparation efficiency and unfavorable to industrialization. For this it is of great importance to obtain highly efficient and specific cyclase.

## 4. CircRNA-based drug delivery systems

### 4.1 Lipid-based nanoparticle (LNP) delivery

Nanoparticles can carry drugs and deliver them to therapeutic targets, and their applications in molecular delivery imaging, therapeutic drugs, and combinations of diagnostic and therapeutic drugs are currently being investigated. Many different sizes and properties of nanoparticle carriers have been designed using nanoparticles, which typically range in size from one-tenth of a nanometer to several hundred nanometers, and these nanoparticle carriers can be made of organic materials such as liposomes, polymers and dendrimers, or inorganic materials such as gold and metal oxides 73. Among them, LNPs are the most advanced nanoparticle carriers that can be used to target specific cells using endogenous or exogenous ligands by encapsulating siRNA, mRNA, and circRNA. Endocytosis of LNPs destabilizes the endosomal membrane and releases nuclear acid cargo such as circRNAs or siRNAs into the cytoplasm. Nanoparticle carriers can solve many of the problems with RNAi molecules, making them less susceptible to degradation and promoting cellular uptake 74-76.

Pain, swelling, fever and drowsiness have been documented in human clinical trials of Pfizer/BioNTech and Moderna vaccines 77-79. The first vaccination's side effects may be linked to significant innate inflammation generated by LNPs, in addition to complement activation-related pseudoallergy (CARPA) reaction 80. Immune responses against cells expressing the vaccine protein or its peptide derivatives, on the other hand, may aggravate side effects following the second immunization 81.

### 4.2 Gold nanoparticles (AuNPs) delivery

AuNP, a well-studied non-viral vector, has been used to deliver circRNAs plasmids due to their high stability, purity, and easy surface modification 82. For example, in a mouse model of dox-induced cardiomyopathy, AuNP delivery of circAmotl1 plasmids reduced apoptosis and improved cardiac function 42. In a mouse trauma model, AuNP delivery of circAmotl1 plasmids promotes the repair of their skin trauma 43. In addition, AuNP delivery of circFoxo3 plasmid promotes tumor cell apoptosis and inhibits tumor growth 44.

Although AuNPs successfully deliver circRNA targets in animal models, it is still largely unclear how safe they are for clinical use. Previous work has demonstrated that toxic effects of AuNPs depend on the size of the particles, with smaller AuNPs causing more deleterious effects 83. Therefore, the properties of AuNPs can be fine-tuned to meet safety requirements, making them a promising delivery system for circRNAs-targeted drugs.

### 4.3 Engineered exosomes

In search of safe and reliable nucleic acid including circRNA delivery vehicles, exosomes have received increasing attention. As early as 1983, Harding's group 84 and Pan's group 85 independently discovered that tiny vesicles smaller than 50 nm secreted by mature reticulocytes were associated with transferrin receptors, and in 1987, Johnstone named such small vesicles as "Exosome "^86^. However, its function was poorly understood by scientists due to the limitations of research tools. It was not until 1996 that Raposo et al. 87 discovered that these tiny vesicles secreted by cells function in antigen presentation during the regulation of immune cells. Exosomes, once thought to be merely "garbage bags" for outward cellular transport, were gradually taken into account by scientists. In the last decade, numerous studies have revealed that exosomes can act as cargo carriers and deliver it to neighboring or distant cells, participating in the regulation of many major diseases. In addition, because of their biocompatibility, exosome can serve as a carrier to deliver small hydrophilic or lipophilic molecules, including some therapeutic drugs. Because of their delivery properties, exosomes can effectively overcome the limitations of poor bioavailability of some drugs when taken orally, reduce the total dose administered, and minimize or remove the side effects caused by the ingestion of certain substances in large doses, and have a promising future in the drug delivery field 88.

Exosomes are also a key mediator of intercellular communication through substances such as specific miRNAs and regulatory proteins they carry 89, 90, where exosomal circRNA can promote cancer progression by promoting cell proliferation, tumor metastasis, and drug resistance. Recent advances indicate that exosomes from chemoresistant colorectal cancer (CRC) cells are rich in ciRS-122. ciRS-122 can bind to miR-122 and upregulates PKM2. PKM2 in turn promotes glycolysis and ATP production. It is thought that up-regulation of PKM2 generates more energy to enable the transporter to excrete drugs from CRC cells. The results indicated that drug-resistant CRC cells deliver ciRS-122 to non-drug-resistant cells via exosomes, making the non-drug-resistant cells resistant to the drug. This study also targeted siRNA to ciRS-122 and thereby enhanced miR-122 levels and decreased PKM2 levels, which improved drug sensitivity in mouse CRC cells^91^. Exosomes can also be used as circRNA delivery vesicles. Li et al. 92 first performed a comprehensive analysis of circRNA in the plasma of acute ischemic stroke patients and found that circSCMH1 was significantly reduced, while the same phenomenon was also found in a stroke mouse model. To enhance circSCMH1 in the brain, they successfully used Lamp2b-RVG modified extracellular vesicles to deliver circSCMH1 to the brain of murine and rhesus stroke models, effectively acting as a recovery agent. The exosome delivery system is similar to the nanoparticle delivery system in that exosomes can effectively protect the delivered drug molecules from degradation and promote cellular uptake without triggering an immune response, while exosomes are more biocompatible than synthetic nanoparticles 93. Currently, research on exosome contents is incomplete, while the large-scale industrial production of exosomes faces great challenges, and there is still a large gap between the use of exosome systems as ideal drug carriers.

## 5. CircRNAs applications

Following the COVID-19 outbreak, the development of mRNA and nucleic acid drugs have gradually become hot topics of interest. mRNA vaccines are a new class of vaccines that consist of mRNA sequences encoding antigens 94. Once expressed in vivo, the target antigen is recognized by the immune system to induce the desired immune response. mRNA vaccines possess a linear single-stranded RNA consisting of a 5' terminal cap, a 5'-untranslated region (UTR), the antigen-coding region, and a 3' polyA tail, which is encapsulated into the organism by LNP 95. Coronavirus disease 2019 (Covid-19) caused by a novel coronavirus, SARS-CoV-2, is a serious global public health emergency 96, 97. Recently developed mRNA vaccines against SARS-CoV-2 have been approved for emergency use 78, 98-102. mRNA vaccines have the advantage that clinical grade mRNA vaccines can be produced rapidly by analyzing viral antigen sequences, while mRNA vaccines are able to respond to viral mutations. However, mRNA vaccines are stored and transported under more demanding conditions, along with potential immunogenic side effects 103.

CircRNA has its own unique advantages in the development of vaccine and nucleic acid drug. Firstly, it is less susceptible to degradation and is more stable than mRNA vaccines and nucleic acid drugs. Secondly, rolling-loop translation requires lower amounts than mRNA and is therefore less toxic. The covalent closed-loop structure enclosed by circRNAs can protect it from degradation by exonucleases and can address the vulnerability of mRNA vaccines to degradation 104. More recently, a circRNA vaccine encoding SARS-CoV-2 RBD has been reported, and the research team successfully induced potent sustained neutralizing antibodies by LNP-encapsulated circRNA-RBD vaccine, and the vaccine was highly heat stable, and its expression was not affected by storage at room temperature for 2 weeks. More importantly, the team successfully induced antibodies that effectively neutralized the B.1.351 mutant strain in mice using a circRNA vaccine encoding the RBD mutant (K417N-E484K-501Y) 105, 106, indicating that the circRNA vaccine also has very promising applications against the SARS-COV-2 mutant strains 107.

Although circRNAs-based vaccines or drugs demonstrated an unprecedented advantage in comparison with mRNA as approved by the enhanced stability, circRNAs drugs are still at an early stage, where there are no mature processes and facilities available for large-scale production of circRNAs. limiting the development of circRNAs drugs. The special structure of circRNAs allows it to translate and produce proteins of different sizes by rolling-loop translation, and also to translate larger proteins using limited nucleotide sequences, which cannot be accomplished by mRNA drugs. However, the uncontrolled accumulation of antigens or proteins can lead to adverse consequences. With the continuous development of circRNAs technology, it is expected that different types of circRNAs drugs can be developed in the future.

## 6. Landscape and future perspectives of circRNAs

CircRNA is another rising star in the RNA family after miRNA, and the understanding of circRNAs is gradually developing. CircRNA was once thought to be an error in the normal splicing process. In recent years, the explosion of circRNA-related research could help in better understanding the mechanism and biological function of circRNA.

Unlike traditional linear RNAs, circRNAs have a closed-loop structure, are less susceptible to degradation, are more stable than mRNAs, and are found in large numbers in the eukaryotic transcriptome. Most circRNAs are composed of exonic sequences that are conserved across species, with tissue and developmental stage specificity of expression. Functionally, circRNAs containing many miRNA binding sites can act as miRNA sponges. miRNAs play a vital role in normal development and homeostasis in vivo as fine-regulators of gene expression, and their dysregulation has been associated with many diseases. CircRNAs play crucial roles mainly by acting as miRNA sponges to bind and inhibit miRNAs. Taking advantage of this feature, the effect of down-regulating oncogenic miRNAs can be achieved by targeting a miRNA that is oncogenic to intervene in cancer progression. CircRNAs can be artificially designed to regulate the amount of miRNAs, achieving efficacy in regulating protein levels and cellular functions.

Current understanding of circRNA is just the tip of the iceberg, and the elucidation of the biological functions and mechanisms of circRNA is still quite distant. Many diseases have been reported to be related to circRNAs. Since circRNA can be detected in the blood and urine of patients due to its stable closed-loop structure, it is expected to be an efficient clinical diagnostic marker in the future, which will be useful for the diagnosis of many diseases and provide new ideas for the development of new targeted therapies and drugs. However, circRNA also has a harmful side. CircRNAs are involved in the production of many cancers and contribute to their development. According to this, circRNA could be a cancer therapeutic target by using siRNA for purposes such as anti-tumour. It is expected that a comprehensive classification and in-depth understanding of circRNAs in all cancers can be carried out in the future to advance circRNAs for cancer therapy.

Still, the challenges are numerous. One of these challenges is low synthetic efficiency. Also, there is a lack of effective circRNA delivery system. The efficiency and safety of nanoparticles and exosomes as drug carriers need to be further confirmed. The delivery efficiency of circRNAs and its targeting can be improved by artificial modification of nanoparticles and exosomes. In the coming years, comprehensive analysis of the functional mechanisms of circRNA remains to be further elucidated, while the development of circRNA with specific targeting will be the key to enhance circRNAs-based therapies.

## Figures and Tables

**Table 1:** CircRNAs database

Name	Link	Description	Literature
CircAtlas	http://circatlas.biols.ac.cn/	Information on circRNA sequences from normal human and many other animal tissues	54
TransCirc	https://www.biosino.org/transcirc/	Greatly expands the information on the evidence for predicting circRNA-encoded proteins, allowing prediction of the potential of specific circRNA-encoded proteins and inference of their translation products	55
riboCIRC	http://www.ribocirc.com/index.html	Predicts the translational potential of circRNAs by pairing Ribo-seq/RNA-seq	56
circRNADb	http://reprod.njmu.edu.cn/circRNAdb	Database of circRNAs that can encode proteins	57
circBase	http://cirbase.org/	Includes information on all identified circRNAs from 6 species, including human and mouse	58
Circbank	http://www.circbank.cn	Comprehensive database of human circRNAs	59
CircPro	http://bis.zju.edu.cn/CircPro	Identifies circRNAs with translation potential	60
CircCode	https://github.com/PSSUN/CircCode	Identifies circRNAs with protein-coding potential with high accuracy	61
Circad	http://clingen.igib.res.in/circad/	Provides disease-associated circRNAs	62
CircInteractome	https://circinteractome.nia.nih.gov/	Predicts binding of circRNAs to RBP or miRNA	63
TSCD	http://gb.whu.edu.cn/TSCD/	Tissue-specific circRNAs in humans and mice	64
MiOncoCirc	https://mioncocirc.github.io/	A clinical tumor sample-based circRNA database	64
circRNADisease	http://cgga.org.cn:9091/circRNADisease/	Provides information on the association between circRNAs and diseases	65
CSCD2	http://geneyun.net/CSCD2/#	A large collection of human cancer-related transcriptome sequencing data to predict potential miRNA-circRNA and RBP-circRNA interactions	66
CircNet 2.0	https://awi.cuhk.edu.cn/~CircNet	Integrates 2,732 samples from 37 cancers and the circRNA-miRNA-gene ceRNA regulatory network	67
VirusCircBase	http://www.computationalbiology.cn/ViruscircBase/home.html	circRNAs in RNA viruses	68
